# FOXM1 Induces a Global Methylation Signature That Mimics the Cancer Epigenome in Head and Neck Squamous Cell Carcinoma

**DOI:** 10.1371/journal.pone.0034329

**Published:** 2012-03-26

**Authors:** Muy-Teck Teh, Emilios Gemenetzidis, Deeviyaben Patel, Rameez Tariq, Ayesha Nadir, Adiam W. Bahta, Ahmad Waseem, Iain L. Hutchison

**Affiliations:** 1 Centre for Clinical and Diagnostic Oral Sciences, Institute of Dentistry, Barts and The London School of Medicine and Dentistry, Queen Mary University of London, London, England, United Kingdom; 2 Centre for Cutaneous Research, The Blizard Institute, Barts and The London School of Medicine and Dentistry, Queen Mary University of London, London, England, United Kingdom; 3 Department of Oral and Maxillofacial Surgery, St Bartholomew's and The Royal London Hospitals, London, England, United Kingdom; Virginia Commonwealth University, United States of America

## Abstract

The oncogene FOXM1 has been implicated in all major types of human cancer. We recently showed that aberrant FOXM1 expression causes stem cell compartment expansion resulting in the initiation of hyperplasia. We have previously shown that FOXM1 regulates *HELLS*, a SNF2/helicase involved in DNA methylation, implicating *FOXM1* in epigenetic regulation. Here, we have demonstrated using primary normal human oral keratinocytes (NOK) that upregulation of *FOXM1* suppressed the tumour suppressor gene *p16^INK4A^* (*CDKN2A*) through promoter hypermethylation. Knockdown of *HELLS* using siRNA re-activated the mRNA expression of *p16^INK4A^* and concomitant downregulation of two DNA methyltransferases *DNMT1* and *DNMT3B*. The dose-dependent upregulation of endogenous *FOXM1* (isoform B) expression during tumour progression across a panel of normal primary NOK strains (n = 8), dysplasias (n = 5) and head and neck squamous cell carcinoma (HNSCC) cell lines (n = 11) correlated positively with endogenous expressions of *HELLS*, *BMI1*, *DNMT1* and *DNMT3B* and negatively with *p16^INK4A^* and involucrin. Bisulfite modification and methylation-specific promoter analysis using absolute quantitative PCR (MS-qPCR) showed that upregulation of *FOXM1* significantly induced *p16^INK4A^* promoter hypermethylation (10-fold, P<0.05) in primary NOK cells. Using a non-bias genome-wide promoter methylation microarray profiling method, we revealed that aberrant *FOXM1* expression in primary NOK induced a global hypomethylation pattern similar to that found in an HNSCC (SCC15) cell line. Following validation experiments using absolute qPCR, we have identified a set of differentially methylated genes, found to be inversely correlated with *in vivo* mRNA expression levels of clinical HNSCC tumour biopsy samples. This study provided the first evidence, using primary normal human cells and tumour tissues, that aberrant upregulation of *FOXM1* orchestrated a DNA methylation signature that mimics the cancer methylome landscape, from which we have identified a unique *FOXM1*-induced epigenetic signature which may have clinical translational potentials as biomarkers for early cancer screening, diagnostic and/or therapeutic interventions.

## Introduction

Understanding the epigenetic mechanism regulating stem-cell fate determination provides fundamental insights into the physiology of tissue regeneration and pathogenesis of cancers. The best studied epigenetic mechanism perturbed during cancer initiation and progression is DNA methylation which chemically adds methyl groups to cytosines at their 5′ positions, predominantly at CpG dinucleotides in the mammalian genomic DNA [1]. DNA methylation involves three key DNA methyltransferases: DNMT1, DNMT3A and DNMT3B. DNMT1 has classically been implicated in maintenance of existing methylated DNA, whereas, DNMT3A and DNTM3B in *de novo* DNA methylation [1]. The heritable nature of DNA methylation enables cells to determine cell potency/fate without changing the primary sequence of genomic DNA. The reversibility of DNA methylation programming renders cell fate specification highly plastic and reversible. Epigenetic reprogramming involving changes in DNA methylation has been implicated in all stages of cancer evolution [2], [3]. It has also been shown that epigenetic reprogramming precedes the initiation of cancer-like stem/progenitor cells [4]. It is now well-accepted that cancer cells exploit the reversible and heritable properties of DNA methylation to perturb the balance between stem/progenitor cell renewal and differentiation thereby promoting cancer initiation and progression [2], [3], [4].

FOXM1 (isoform B) was first found to be a downstream target of an oncogenic Sonic Hedgehog signalling pathway via a glioma family zinc finger transcription factor 1 (Gli1) in basal cell carcinomas [5]. Subsequent studies revealed that FOXM1 was ubiquitously upregulated in the majority of human cancers [6], [7] which include brain, liver, breast, lung, stomach, pancreas, colon, kidney, bladder, prostate, testis, ovary, uterus, cervix, blood (acute myeloid leukaemia), cutaneous melanoma, head and neck squamous cell carcinomas [8], [9].

In the quest to understand the oncogenic mechanism of FOXM1, we have recently shown that FOXM1 induces cancer initiation by promoting adult human epithelial stem/progenitor cell renewal and by antagonising differentiation [10]. Others have demonstrated that FOXM1 plays a key role in maintaining stem/progenitor cell renewal through pluripotency genes including *Oct4*, *Nanog*, *Sox2* and *Bmi1* [11], [12]. Our previous work identified a FOXM1 downstream target *HELLS* [8], a human embryonic stem cell factor/lymphoid-specific SNF2/helicase involved in chromatin remodelling and DNA methylation [13], [14], implicating FOXM1 in epigenetic regulation during stem/progenitor cell renewal [8], [10]. However, it was unclear whether FOXM1 has a role in epigenetic regulation. In this study, using primary normal human oral keratinocytes and head and neck squamous cell carcinoma (HNSCC) tumour cell lines and tumour biopsy tissues, we investigated the role of FOXM1 in the regulation of gene promoter methylation at both single gene and genome-wide levels. This led to the first evidence in normal primary human oral epithelial cells that FOXM1 induces a methylation landscape resembling a cancer epigenome found in HNSCC tumour tissues.

## Methods

### Clinical Tissues

The use of human tissue in this study has been approved by our host institutions (Barts & the London NHS Trust and the School of Medicine & Dentistry, Queen Mary University of London) and the UK National Research Ethics Committee. All clinical samples, which were surplus to diagnosis, were collected according to local ethical committee-approved protocols and written informed patient consent was obtained from all participants. Pairs of normal margin and HNSCC tumour core tissue biopsies were histopathological pre-validated by our collaborating pathologists prior to use for this study. Fresh biopsy tissue samples were preserved in RNA*Later* (Cat# AM7022, Ambion, Applied Biosystems, Warrington, UK) and stored short-term at either 4°C (1–2 days) or −20°C (up to 1 week) prior to transportation and subsequent long-term storage at −80°C until use.

### Cell culture

All primary normal human oral keratinocytes (OK355, HOKG, OK113, NOK, NOK1, NOK3, NOK16 and NOK376) were extracted from normal oral mucosa tissues donated by healthy disease-free individuals undergoing wisdom tooth extraction and cultured as previously described [8], [15]. Oral dysplastic precancer cell lines (OKF6/T [16], POE9n [17], DOK [18], D19 [19], D20 [19]) and oral SCC cell lines (SCC4 [20], SCC9 [20], SCC15 [20], SCC25 [20], SqCC/Y1 [21], UK1 [22], VB6 [22], CaLH2 [22], CaDec12 [22], 5PT [22], H357 [22]), SVpgC2a [23] and SVFN1-8 [8] were all well-established cell lines cultured as described previously [8], [10], [15].

### Immunoblotting

Protein extraction and separation on SDS-PAGE gels and immunoblotting was performed as previously described (5). A mouse monoclonal antibody for p16^INK4A^ (1∶2000 dilution; Cat# 551154, BD Biosciences) and a rabbit polyclonal anti-GAPDH (1∶20,000 dilution; Cat# 9485, Abcam) were used for immunoblotting.

### RNA interference

Pre-validated gene-specific siHELLS (ON-TARGETplus SMARTpool HELLS, Cat# L-017444-09,10,11,12), control siCTRL (ON-TARGETplus Non-targeting Pool, Cat# D-001810-10-05) and siRNA transfection reagent (DharmaFECT 1, Cat# T-2001-02) were purchased from Dharmacon, Thermo Fisher Scientific. An initial dose-response experiment was performed according to manufacturer's instructions to determine the optimum transfection efficiency. siRNA at 10 nM (48-hour incubation) was found to be the optimum final concentration which was therefore used in all subsequent experiments. The effect of gene silencing was validated by quantification of the target gene mRNA expression (*HELLS*) by absolute reverse transcription qPCR.

### Retroviral transduction

**Figure 1:**
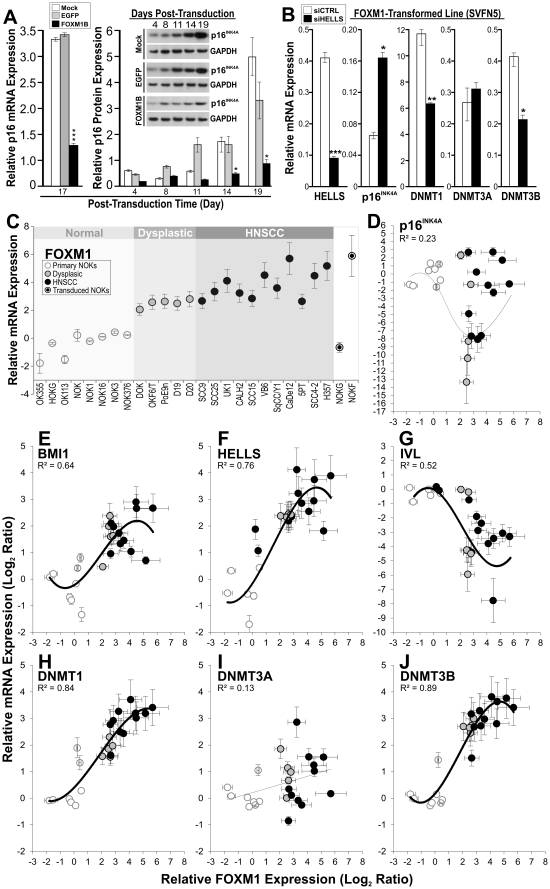
Upregulation of FOXM1 suppressed *p16^INK4A^* expression in primary human oral keratinocytes. (**A**) FOXM1 significantly supresses *p16^INK4A^* mRNA and protein expression (inset figure) in primary normal human keratinocytes. GAPDH was used as a control for protein loading. Control cells (mock-transduced with empty retroviral particles or EGFP-transduced) did not show significant suppression of p16^INK4A^ expression. (**B**) Knockdown of a FOXM1-target gene *HELLS*, which regulates genome-wide methylation [14], induced *p16^INK4A^* and simultaneously suppressed *DNMT1* and *DNMT3B*, but not *DNMT3A* mRNA expression in a FOXM1-transformed malignant cell line (SVFN5) expressing constitutive levels of endogenous *HELLS* [8]. Each bar represents a mean ± SEM of triplicate transfection (48 h) with either siCTRL or siHELLS. *P<0.05, **P<0.01 and ***P<0.001 indicate the level of statistical significance compared to controls. (**C**) Endogenous *FOXM1* (isoform B) mRNA expression levels in 8 strains of primary human normal oral keratinocytes, 5 dysplastic and 11 HNSCC cell lines. Total *FOXM1* mRNA expression levels were measured in the EGFP and FOXM1-transduced NOK (NOKG and NOKF), respectively. (**D**–**J**) Third-order polynomial regression analyses were performed to obtain the R^2^ coefficient of determination values which indicate the significance of co-expression between each gene with *FOXM1* across the 24 cell strains/lines indicated in panel C.

Retroviral supernatant and transduction procedures were performed using our established protocols [8], [10], [15]. Equal levels of *EGFP* and *FOXM1* (isoform B) expression were achieved by serial retroviral supernatant titration experiment and subsequently *EGFP* plasmid copy number confirmed by qPCR using genomic DNA extracted from transduced cells according to our previously established method [15]. The levels of ectopic *FOXM1* expression in the primary keratinocytes were titrated to replicate levels found in cancer cells as reported previously [8], [10], [15] (see Figure 1C). Transduced cells were cultured for 3–5 days to allow transgene expression prior to experiment.

### Nucleic Acids Preparations from Tissues and Cells

All tissue biopsies were digested by proteinase K (Cat# 03115887001, Roche Diagnostics Ltd., England, UK) prior to simultaneous mRNA extraction (Dynabeads mRNA Direct kit, Cat# 610.12, Invitrogen) and genomic DNA (gDNA) extraction (by standard phenol∶chloroform method on mRNA-depleted lystates). mRNA was immediately reverse transcribed into cDNA (Transcriptor cDNA Synthesis kit, Cat# 04897030001, Roche Diagnostics). gDNA were fragmented by *MseI* digestion (37°C, 16 h) prior to enrichment for CpG-methylated DNA using a MBD2b/MBD3L1-conjugated magnetic bead-based system according to manufacturer's protocol (MethylCollector Ultra kit, Cat# 55005, Active Motif Europe, Belgium).

### Genome-wide Promoter Methylation Profiling

According to manufacturer's protocol and requirements, input *MseI*-digested gDNA and methylation-enriched DNA from each cell sample (NOKG, NOKF and SCC15) were amplified to generate 6 µg DNA using WGA2 GenomePlex (Sigma) prior to microarray experiments performed by Roche NimbleGen microarray service using Human DNA Methylation 3x720K CpG Island Plus RefSeq Promoter Array (Cat# 05 924 600 001; NimbleGen System, Reykjavik, Iceland) based on genome built HG18, with promoter upstream/downstream tilling of −2.44/+0.61 kb, covering a total of 27,728 CpG islands across the whole genome (GEO Platform: GPL14361). Microarray data generated in this study is MIAME compliant and has been deposited in a MIAME compliant database at Gene Expression Omnibus repository (GEO Series accession number: GSE31767).

### Real-time absolute quantitative PCR

Standard curve-based real-time absolute quantitative PCR were performed using SYBR Green I Master (Cat# 04887352001, Roche Diagnostics Ltd, England, UK) in the 384-well LightCycler 480 qPCR system (Roche) according to our established protocols [8], [9], [15] which are MIQE compliant [24]. Methylation-specific PCR conditions were performed as described previously [25], [26]. All primers used in this study are listed in Figure S1. Previously validated isoform B-specific FOXM1 primers were used to specifically quantify FOXM1 (isoform B) mRNA expression in this study [8]. All target genes were normalised to two stable reference genes (YAP1 and POLR2A) previously validated to be amongst the most stable reference genes across a wide variety of primary human oral cells, dysplastic and HNSCC cell lines [8].

## Results and Discussion

Given our previous finding that FOXM1 (isoform B) promoted stem/progenitor cell renewal through perturbing the differentiation pathway [10], we initially questioned the involvement of a tumour suppressor gene *p16^INK4A^* (*CDKN2A*) given that it has been shown to regulate epithelial stem/progenitor cell differentiation [27] and it is the most commonly inactivated gene in cancer [28]. Here, we showed that ectopic *FOXM1* expression suppressed both mRNA and protein expression of p16^INK4A^ in primary human oral keratinocytes (Figure 1A). Unfortunately, as reported previously silencing endogenous *FOXM1* expression causes cell cycle arrest [29] which precluded further experiments using RNAi on the notoriously sensitive primary human oral keratinocytes [8], [10]. Nevertheless, our *FOXM1* overexpression experiments conclusively showed that *FOXM1* upregulation suppressed *p16^INK4A^* gene expression in primary human oral keratinocytes. This is in agreement with previous findings that *FOXM1* suppresses the senescence pathway mediated by *p16^INK4A^* in cancer cells [30].

Inactivation of *p16^INK4A^* gene expression could be a result of a number of mechanisms including gene deletion and promoter hypermethylation. Given that FOXM1 targets *HELLS* which regulates DNA methylation [13], [14], we hypothesised that FOXM1 may be suppressing *p16^INK4A^* expression through promoter hypermethylation via *HELLS*. To test this, we knockeddown *HELLS* by siRNA in an HNSCC cell line SVFN5, a FOXM1-induced transformed oral buccal keratinocyte SVpgC2a line [8], that expresses high levels of endogenous *HELLS* and low levels of *p16^INK4A^*. This causes re-activation of the mRNA expression of *p16^INK4A^* (Figure 1B) and concomitant downregulation of two DNA methyltransferases *DNMT1* and *DNMT3B* but no effect on *DNMT3A* expression. The fact that *p16^INK4A^* inhibition could be reactivated argues against gene deletion as a mechanism for *p16^INK4A^* inactivation. Our results are consistent with previous findings that *HELLS* interacts with *DNMT1* and *DNMT3B* [31] to suppress *p16^INK4A^* gene expression [32] through epigenetic modifications.

To further validate that the expression of *FOXM1*, *HELLS* and *p16^INK4A^* genes correlate with cancer progression and whether there are any associations with genes involved in DNA methylation, we measured the endogenous mRNA expression levels of *FOXM1*, *p16^INK4A^*, *HELLS*, *BMI1*, involucrin (*IVL*, a differentiation marker has been shown to be negatively regulated by *FOXM1* [10]) and 3 key DNA methyltransferases (*DNMT1*, *DNMT3A*, *DNMT3B*) in a panel of 24 cell strains/lines consisting of 8 strains of primary normal human oral keratinocytes (from normal oral mucosa tissues), 5 dysplasia and 11 HNSCC cell lines.

In agreement with previous findings [8], [10], *FOXM1* showed dose-dependent upregulation during tumour progression from dysplasia to HNSCC (Figure 1C). Across the panel of 24 cell strains/lines, we have found that the endogenous mRNA expression of *FOXM1* correlated inversely with *p16^INK4A^* but correlation efficiency was weak (R^2^ = 0.23, Figure 1D). The downregulation of *p16^INK4A^* expression was found to be more pronounced in dysplastic compared to HNSCC cell lines. Such *p16^INK4A^* expression pattern is in complete agreement with *in vivo p16^INK4A^* protein expression pattern found in oral dysplasia and SCC tissues [33]. Consistently, *BMI1*, a polycomb group oncogene which is an upstream regulator of *p16^INK4A^* gene [34] and also a downstream target of FOXM1 [12], [30], showed positive co-expression with *FOXM1* (R^2^ = 0.64, Figure 1E) but weak inverse correlation with *p16^INK4A^* (R^2^ = 0.42, data not shown) supports the evidence that *p16^INK4A^* expression is independently regulated by *BMI1* during oral carcinogenesis [35]. The discordant expression levels between *FOXM1* and *p16^INK4A^* in cancer cells may be due to the fact that *p16^INK4A^* can be deregulated through a number of different mechanisms, such as inactivating mutation (may result in upregulation due to feedback mechanism), gene deletion, gene amplification (of functional gene but defective downstream signalling), promoter hypermethylation, etc. This may result in varying *p16^INK4A^* expression independent of *FOXM1* levels in the “cancer” cell lines. Hence, whilst *FOXM1* can induce promoter hypermethylation of *p16^INK4A^* in “normal” cells, such effect may be perturbed in “cancer” cells.

Expression of *DNMT1* (R^2^ = 0.84; Figure 1H) and *DNMT3B* (R^2^ = 0.89; Figure 1J), but not *DNMT3A* (R^2^ = 0.13; Figure 1I), showed significant positive co-expression with *FOXM1* which are in agreement with our findings above (Figure 1B) that silencing the *FOXM1*-downstream target *HELLS* led to concomitant downregulation of *DNMT1* and *DNMT3B* but no effect on *DNMT3A* expression. It is unclear why *DNMT3A* was not affected. Published literature indicates that although both *DNMT3A* and *DNMT3B* are involved in *de novo* methyltransferase activity, they serve non-overlapping roles [1]. Nevertheless, the involvement of both *DNMT1* and *DNMT3B* implicates a role for *FOXM1* and *HELLS* in triggering both maintenance and *de novo* DNA methylation activities [1]. Expectedly, *HELLS* were positively (R^2^ = 0.76, Figure 1F) and *IVL* were negatively (R^2^ = 0.52, Figure 1G) correlated with *FOXM1* as shown previously [8], [9], [10]. Collectively, these results provide the first evidence in human cells that *FOXM1* may be acting through *HELLS*, *DNMT1* and *DNMT3B* to suppress *p16^INK4A^* gene expression. Given that *HELLS*, *DNMT1* and *DNMT3B* have been previously shown to modulate *p16^INK4A^* promoter methylation [31], [32], we hypothesised that *FOXM1* may be triggering *p16^INK4A^* gene silencing through promoter hypermethylation.

**Figure 2:**
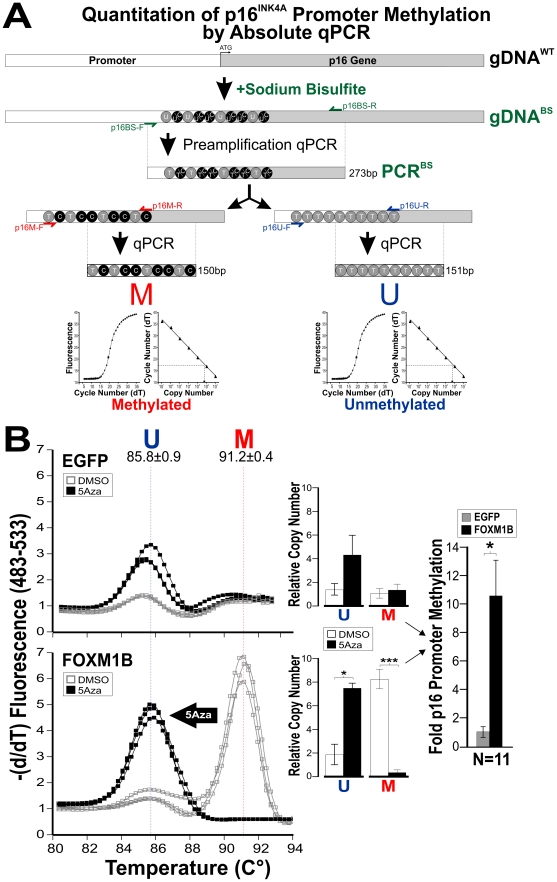
*FOXM1* induces promoter hypermethylation of *p16^INK4A^* gene in primary human oral keratinocytes. (**A**) Bisulfite modification and methylation specific absolute qPCR for the quantification of *p16^INK4A^* promoter methylation status. Genomic DNA was first treated with sodium bisulfite prior to PCR pre-amplification of the promoter region of *p16^INK4A^* (PCR^BS^, 273 bp). Methylation specific (p16M-R/F) and methylation-independent (p16U-F/R) primers were then used to quantify the relative levels of methylated and unmethylated products within the PCR^BS^ sample using standard-curve based absolute qPCR method for each product, respectively. Melting analysis was performed to validate the qPCR specificity in detecting the two M and U products. (**B**) Bisulfite conversion and methylation specific qPCR were performed to measure the relative levels of unmethylated (U, melting temperature at 85.8°C) and methylated (M, 91.2°C) in either EGFP- or FOXM1-transduced primary NOK treated with either vehicle (DMSO) or 5Aza (1 µM, 3-day incubation with fresh drug replenishment daily). A total of n = 11 replicates from at least 4 independent experiments were performed. Statistical t-test significance notations *P<0.05 and ***P<0.001.

To investigate promoter CpG DNA methylation, we quantified the level of *p16^INK4A^* promoter methylation using bisulfite modification and methylation-specific quantitative PCR (MS-qPCR; Figure 2A and Figure S1). Overexpression of *FOXM1*, but not *EGFP*, was found to induce *p16^INK4A^* promoter hypermethylation (P<0.05) which was significantly reversed (P<0.001) by a DNA demethylating agent 5-aza-2′-deoxycytidine (5Aza) in primary human oral keratinocytes (Figure 2B). These results confirmed a role of *FOXM1* in suppressing *p16^INK4A^* expression through promoter hypermethylation. In support for *FOXM1* in initiating oncogenesis through the inhibition of *p16^INK4A^*, it has been shown that epigenetic silencing of *p16^INK4A^* induces cellular immortalisation in mouse embryonic fibroblasts [36]. Furthermore, our previous finding that *FOXM1* expression co-expressed with an epithelial stem cell marker ΔNp63α in the proliferating stem/progenitor oral keratinocyte subpopulation [10], and that ΔNp63α has been shown to target *HELLS* to induce squamous cell carcinoma formation in mice [37], together suggest a possible role for *FOXM1* (via *HELLS*) in triggering oncogenesis through silencing *p16^INK4A^*. The exact oncogenic mechanism is beyond the scope of this study. Nevertheless, our current data providing the first evidence that *FOXM1* is able to induce promoter hypermethylation at a single gene level offers a glimpse of possibility that aberrant upregulation of *FOXM1* may perturb the epigenetic regulation of DNA methylation at genome-wide level.

**Figure 3:**
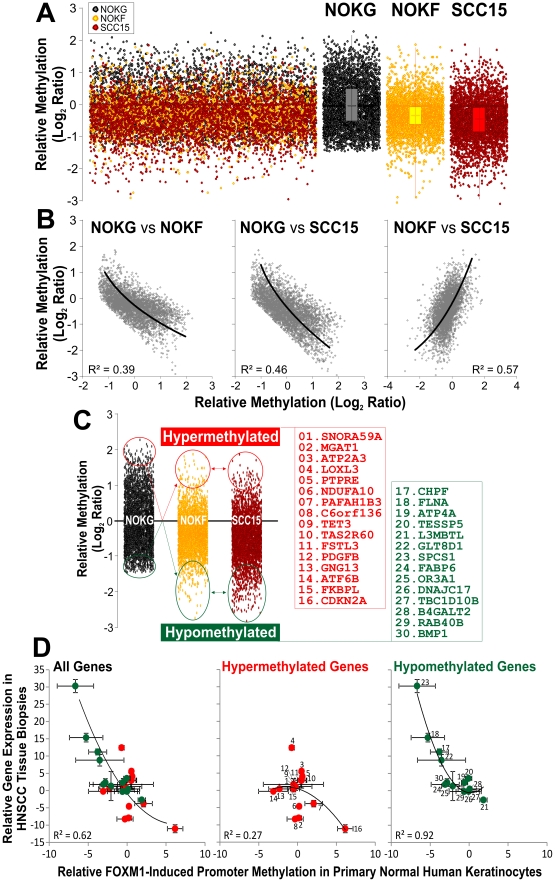
Upregulation of *FOXM1* (isoform B) induces a global shift in methylation pattern that mimics the cancer epigenome. (**A**) Genome-wide promoter microarray analysis of primary normal oral human keratinocytes expressing either *EGFP* (NOKG, black dots) or *FOXM1* (NOKF, yellow dots) and an established squamous cell carcinoma cell line (SCC15, red dots). Each dot represents a single gene. (**B**) A non-linear 2^nd^ order polynomial regression analyses were performed on the relative methylation patterns between NOKG vs NOKF (inverse correlation), NOKG vs SCC15 (inverse correlation) and NOKF vs SCC15 (positive correlation). (**C**) Gene selection criteria for differentially methylated genes between control (NOKG) and tests groups (NOKF and SCC15). 100-most hypermethylated and 100-most hypomethylated genes were inversely matched with differentially methylated genes from NOKF and SCC15. The adjacent gene lists show the shortlisted FOXM1-induced (also found in SCC15) differentially hypermethylated (red) and hypomethylated (green) genes compared to control NOKG cells. The CDKN2A (encodes *p16^INK4A^*) gene, its promoter known to be hypermethylated in HNSCC, was included as a positive control for promoter hypermethylation. (**D**) Clinical tumour tissue sample correlation between the relative levels of methylation and gene expression of each shortlisted gene in a cohort of 10 patients with paired normal margin and HNSCC tumour tissue samples. Each dot represents mean ± SEM of each gene. Vertical error bars were derived from relative gene expression of 10 margin-tumour tissue pairs and horizontal error bars were derived from relative promoter methylation of 3 independent primary NOK (NOKG/NOKF) experiments. Correlation coefficient (R^2^) of a non-linear 2^nd^ order polynomial regression analyses were performed on all 30 candidate genes (left panel), 16 hypermethylated genes (middle panel) or 14 hypomethylated genes (right panel), respectively.

We and others have previously established a central role for *FOXM1* in the maintenance of genome stability whereby aberrant *FOXM1* expression causes global genomic instability [8], [15], [38]. Furthermore, the findings that *FOXM1* targets an epigenetic/stem cell modulator *HELLS* during cancer initiation [8], [14] and *FOXM1* directly induces *p16^INK4A^* promoter hypermethylation (Figure 2) prompted us to hypothesise that aberrant upregulation of *FOXM1* perturbs the methylome. To test this hypothesis, we performed a non-bias genome-wide promoter methylation microarray profiling on primary normal oral human keratinocytes (NOK) either overexpressing a control gene *EGFP* (NOKG) or *FOXM1* (NOKF) (see Figure 1C for *FOXM1* gene expression levels of NOKG and NOKF cells), and also on an HNSCC cell line (SCC15). SCC15 was chosen in this study as a positive control because the promoter of *p16^INK4A^* gene (CDKN2A) has been previously shown to be hypermethylated and could be reactivated by 5Aza [39], hence allowing us to validate the methylation array data. *FOXM1* was found to induce a global hypomethylation pattern similar to that found in the HNSCC cell line, compared to control NOK cells expressing *EGFP* (Figure 3A). Comparing the methylation patterns by regression correlation analyses amongst the three cell types (NOKG, NOKF and SCC15), only NOKF vs SCC15 gave a positive correlation pattern, whereas NOKF or SCC15 each produced an inverse correlation with the control NOKG (Figure 3B). This indicates that overexpression of *FOXM1*, but not *EGFP*, induces a methylation landscape similar to that found in SCC15. Both global hypomethylation and focal hypermethylation (affecting individual genes) are typical methylation patterns found in cancer [2], [3]. The fact that upregulation of FOXM1 induces these methylation patterns in “normal” cells indicates that aberrant expression of FOXM1 is changing the methylation landscape towards those of cancer. The consequence of global hypomethylation has been shown to cause genomic instability [2], [3], this may provide a mechanism for our previous findings that aberrant FOXM1 expression causes genomic instability in primary normal human keratinocytes [8], [15]. Although global hypomethylation appeared to be the dominating effect, it has been shown that focal hypermethylation silencing key tumour suppressor genes (eg. *p16^INK4A^*) also plays important role in oncogenesis [2], [3].

To validate our hypothesis that *FOXM1*-orchestrated a methylation signature that mimics a cancer methylome, differentially methylated genes (100 most hypomethylated and 100 most hypermethylated) were initially selected for inverse comparisons between NOKG and NOKF/SCC15, and a subset of 30 consensus genes, shared between NOKF and SCC15 cells, with opposing methylation status to NOKG control cells, were subsequently shortlisted for further analyses (Figure 3C). If these candidate *FOXM1*-induced differentially methylated genes were indeed an epigenetic signature of cancer, we hypothesised that HNSCC tumour tissues should retain an inverse *in vivo* mRNA expression signature of these candidate genes. To verify this, we performed absolute qPCR to quantify each of the 30 candidate genes: i, the relative levels of promoter DNA methylation of each gene in NOKG vs NOKF cells, and, ii, the relative mRNA expression levels in paired normal margin vs HNSCC tumour tissue samples. Correlation regression analyses of the 30 candidate genes showed an inverse relationship (R^2^ = 0.62; Figure 3D, left panel) between gene expression of HNSCC tumour tissues and DNA methylation of NOKF cells.

Interestingly, hypomethylated genes showed significantly higher inverse correlation pattern (R^2^ = 0.92; Figure 3D, right panel) than the hypermethylated genes (R^2^ = 0.27; Figure 3D, middle panel). This suggests that promoter hypomethylation exhibited a stronger effect on transcriptional activation compared to promoter hypermethylation on transcriptional repression. One explanation could be that it may be easier to detect transcriptional activation following promoter hypomethylation as opposed to detecting transcriptional repression which depends on whether the genes were activated prior to hypermethylation. Our results indicate that hypo/hypermethylation may not be a simple symmetrical on/off switch for gene transcription. Further studies are required to delineate the transcriptional mechanisms regulated by promoter DNA methylation/demethylation.

Of the list of 15 novel *FOXM1*-induced hypermethylated genes (Figure 3D, middle panel), 4 genes (*C6orf136*, *MGAT1*, *NDUFA10* and *PAFAH1B3*) had significantly downregulated mRNA expression levels in HNSCC tumours, along with the positive control *p16^INK4A^* (*CDKN2A*). Little published gene information was available for *C6orf136*. *MGAT1* [mannosyl (alpha-1,3-)-glycoprotein beta-1,2-N-acetyl-glucosaminyltransferase] has been implicated in glycerolipid metabolism [40]; *NDUFA10* (NADH dehydrogenase (ubiquinone) 1 alpha subcomplex, 10, 42 kDa) in mitochondrial metabolism [41] and PAFAH1B3 (platelet-activating factor acetylhydrolase 1b, catalytic subunit 3, 29 kDa) in brain development [42] and spermatogenesis [43]. Given that their gene expressions were suppressed through promoter hypermethylation in tumour tissues, we speculate that they may be tumour suppressor genes. However, their roles in tumourigenesis remained to be investigated.

Of the list of 14 novel *FOXM1*-induced hypomethylated genes (Figure 3D, right panel), 4 genes (*SPCS1*, *FLNA*, *CHPF* and *GLT8D1*) had significantly upregulated mRNA expression levels in HNSCC tumours. *FLNA* (filamin A, alpha), an actin-binding protein involves in cytoskeletal/membrane remodelling and cellular motility [44], [45], has been implicated in melanomagenesis [44], [46], prostate [47], [48], [49], breast [50], lung [51], liver [52] and ovarian cancers [53]. *CHPF* (chondroitin polymerizing factor), involved in extracellular matrix regulation [54], has recently been implicated in colorectal cancer [55]. SPCS1 (signal peptidase complex subunit 1 homolog) and *GLT8D1* (glycosyltransferase 8 domain containing 1) are located adjacent to each other at chromosome 3p21.1. Given that their gene expressions were upregulated in tumour tissues, we speculate that they may be oncogenes. However, their roles in oncogenesis remained to be investigated.

Collectively, these results confirmed that aberrant expression of FOXM1 triggers genome-wide methylomic alterations that mimic the *in vivo* cancer methylome of HNSCC tumour tissues. We speculate that this may be a mechanism exploited by FOXM1 to induce progenitor/stem cells expansion [10] through methylome reprogramming to antagonise differentiation.

In summary, we have shown for the first time that aberrant upregulation of a single oncogene *FOXM1* in primary normal human oral epithelial cells orchestrated a cancer-like methylome landscape, from which we have identified a unique set of FOXM1-induced differentially methylated genes. We further provided evidence that their *in vivo* gene expression signatures were retained in HNSCC tumour tissues. Given that epigenetic alteration precedes gene expression, we speculate that the FOXM1-induced differentially methylated genes have strong potential as epigenetic biomarkers for early cancer screening, diagnostic, prognostic and/or therapeutic interventions.

## Supporting Information

Figure S1 **Absolute qPCR primers.** (A) Nucleotide sequence of the bisulfite treated promoter region of p16INK4A and their respective primer sequences used in this study. Details of qPCR conditions were performed according to published methods [25], [26]. (B) qPCR primer sequences of the 30 candidate FOXM1-induced differentially methylated genes. Colour shaded loci indicate that the genes were adjacent or nearby. Promoter CpG islands (CGI) for each gene are annotated as either ‘S’ (sense strand), ‘AS’ (antisense strand) or ‘-’ (no CGI within promoter region). All primer pairs produce a single melting peak. Standard curves were generated for each gene for absolute quantification of unknown samples according to protocols described previously [8].
(PDF)
