# Supplementary material for: FOXM1 Induces a Global Methylation Signature That Mimics the Cancer Epigenome in Head and Neck Squamous Cell Carcinoma
Source: PLoS One. 2012 Mar 26;7(3):e34329. doi: 10.1371/journal.pone.0034329 (PMC3312909; doi:10.1371/journal.pone.0034329)
Supplement: Figure S1 — Absolute qPCR primers. (A) Nucleotide sequence of the bisulfite treated promoter region of p16INK4A and their respective primer sequences used in this study. Details of qPCR conditions were performed according to published methods [25], [26]. (B) qPCR primer sequences of the 30 candidate FOXM1-induced differentially methylated genes. Colour shaded loci indicate that the genes were adjacent or nearby. Promoter CpG islands (CGI) for each gene are annotated as either ‘S’ (sense strand), ‘AS’ (antisense strand) or ‘-’ (no CGI within promoter region). All primer pairs produce a single melting peak. Standard curves were generated for each gene for absolute quantification of unknown samples according to protocols described previously [8]. (PDF) [file pone.0034329.s001.pdf]

Figure S1

A

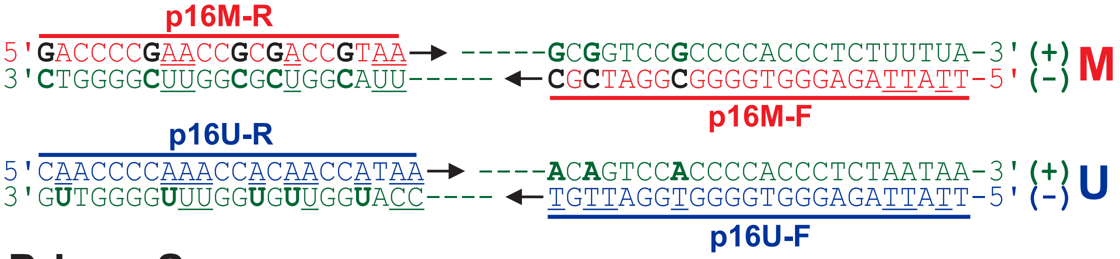

Primer Sequences

C/G - Methylated CpG
A/T - Unmethylated CpG

p16BS-F 5'-GAAGAAAGAGGAGGGGTTGG-3'
p16BS-R 5'-CTACAAACCCTCTACCCACC-3'
p16M-F 5'-TTATTAGAGGGTGGGGCGGATCGC-3'
p16M-R 5'-GACCCCGAACCGCGACCGTAA-3'
p16U-F 5'-TTATTAGAGGGTGGGGTGGATTGT-3'
p16U-R 5'-CAACCCCAAACCACAACCATAA-3'

Georgiou et al., (2007) Eur J Cancer Prev 16:396–402
Palmisano et al., (2000) Cancer Res 60:5954-5958

B

Hypermethylated

	Loci	CGI	Primer	bp	Primer Sequence
1	1p36	-	SNORA59-F	81	gcccagggtatgttcacg
			SNORA59-R		cgttgtgaggcagttaatgttc
2	5q35	AS	MGAT1-F	128	tgccctgctgctcctcttct
			MGAT1-R		ccacctcggcgtcttggg
3	17p13	AS	ATP2A3-F	138	aatatcacatcgggcaaagc
			ATP2A3-R		ccgtccaaactcgtccag
4	2p13	S	LOXL3-F	103	acgggccctgagaagaag
			LOXL3-R		caccagctcgctgtatctcc
5	10q26	AS	PTPRE-F	71	caaagaaagtccattgtacataacaaa
			PTPRE-R		gtgaaaggaacaatggtgtcg
6	2q37	AS	NDUFA10-F	77	cacaaaaagcaatccatcctg
			NDUFA10-R		aggtttccaaacatccagaa
7	19q13	S	PAFAH1B3-F	106	cctgcttctgcgtctgct
			PAFAH1B3-R		ggaaggagagtttaatgttgtgg
8	6p21	-	C6orf136-F	74	gtggaggcagccaagaatc
			C6orf136-R		gcatgagatggaaaggaagg
9	2p13	-	gTET3-F	110	aggtgcacagagtgcgagt
			gTET3-R		gcctattgctctgctcttgc
10	7q35	S	gTAS2R60-F	76	ccatggatgctcttcagctc
			gTAS2R60-R		acattctgtggttgcctatgaa
11	19p13	AS	gFSTL3-F	95	aaaagtgcccctaggttggt
			gFSTL3-R		cttgagtctttatttccttggtgag
12	22q13	AS	gPDGFB-F	93	tcccacctactgcactttcc
			gPDGFB-R		aaaggaaagcccccaaaaat
13	16p13	AS	gGNG13-F	102	ggccccactcacaacatct
			gGNG13-R		aggcgtggtctcacaggata
14	6p21	AS	gATF6B-F	89	tggtgagctgctgcatattt
			gATF6B-R		acttcccttgtcccacctg
15	6p21	AS	gFKBPL-F	123	tgctagggcagcctcagt
			gFKBPL-R		ctttttccaggttcccaagg
16	9p21	AS	p16-F	94	tgcccaacgcaccgaatag
			p16-R		caccagcgtgtccaggaag

Hypomethylated

	Loci	CGI	Primer	bp	Primer Sequence
17	2q35	AS	CHPF2-F	100	gggctgggctgtagaagagt
			CHPF2-R		ttaaaggcagggagcttcg
18	Xq28	AS	FLNA2-F	88	aggacaagggggagtacaca
			FLNA2-R		cagactcagggcaccacaa
19	19q13	AS	ATP4A-F	92	gaaggccgagaactatgagc
			ATP4A-R		ccgccttcttcttcttgct
20	3p21	-	TESSP5-F	101	caatggagccttctcaggtc
			TESSP5-R		ggaggaaaaggtagggaggtc
21	20q13	S	L3MBTL-F	84	ggctggtgtagcttggagtg
			L3MBTL-R		cctctcttcgcctcatgc
22	3p21	AS	gGLT8D1-F	86	gcgacgctctagcggtta
			gGLT8D1-R		cgagcacacttgccctct
23	3p21	S	gSPCS1-F	80	cgcgcaagtactgtcaagg
			gSPCS1-R		gaagtgttcgccgtcagtg
24	5q33	-	gFABP6-F	80	tgacctatgagcgcgtgagc
			gFABP6-R		ttttattggtgggtttgtagctc
25	17p13	-	gOR3A1-F	93	agctgcagtcctgcgaat
			gOR3A1-R		ccatagaatatggcaaccacag
26	15q15	AS	gDNAJC17-F	93	gatgcagcaggaagaccag
			gDNAJC17-R		aaatttattggtgacgttgaagaa
27	16p11	AS	gTBC1D10B-F	92	gctcagctggggtctctgt
			gTBC1D10B-R		cacccctgggatgacaac
28	1p24	S	gB4GALT2-F	112	ggggcttggatcagtaagtct
			gB4GALT2-R		ctaaagcacccacacaagtttct
29	17q35	AS	gRAB40B-F	76	agggaagaaaatgccaaagat
			gRAB40B-R		agctcttccttgacctgtcg
30	8p21	S	gBMP1-F	81	attcctcaccaagctcaacg
			gBMP1-R		tgccagatgcagttcttgtt

Figure S1. Absolute qPCR primers. **(A)** Nucleotide sequence of the bisulfite treated promoter region of p16[INK4A] and their respective primer sequences used in this study. Details of qPCR conditions were performed according to published methods (Georgiou et al., 2007; Palmisano et al., 2000). **(B)** qPCR primer sequences of the 30 candidate FOXM1-induced differentially methylated genes. Colour shaded loci indicate that the genes were adjacent or nearby. Promoter CpG islands (CGI) for each gene are annotated as either 'S' (sense strand), 'AS' (antisense strand) or '-' (no CGI within promoter region). All primer pairs produce a single melting peak. Standard curves were generated for each gene for absolute quantification of unknown samples according to protocols described previously (Gemenetzidis et al., 2009).
